# Supplementary material for: Dynalign II: common secondary structure prediction for RNA homologs with domain insertions
Source: Nucleic Acids Res. 2014 Nov 21;42(22):13939–48. doi: 10.1093/nar/gku1172 (PMC4267632; doi:10.1093/nar/gku1172)
Supplement: SUPPLEMENTARY DATA [file supp_gku1172_nar-02021-z-2014-File012.zip › manual/Text/dot2ct.html]

RNAstructure Command Line Help: dot2ct


|  |  |  |
| --- | --- | --- |
|  | RNAstructure Command Line Help dot2ct | - Contents - Index |
| dot2ct is used to generate a CT file from a dot bracket file. The dot bracket structure can be regenerated from the CT structure by using the ct2dot interface. USAGE: dot2ct <bracket file> <ct file> Required parameters: <bracket file> \| The name of a dot bracket file containing the structure to convert.; <ct file> \| The name of a CT file to which output will be written. Options which don't require added values: -h, -H, --help \| Display the usage details message. Options which require added values: NONE References: 1. Reuter, J.S. and Mathews, D.H. "RNAstructure: software for RNA secondary structure prediction and analysis." *BMC Bioinformatics*, 11:129. (2010). | | |
| Visit The Mathews Lab RNAstructure Page for updates and latest information. | | |
